# Autonomous Crack Detection for Mountainous Roads Using UAV Inspection System

**DOI:** 10.3390/s24144751

**Published:** 2024-07-22

**Authors:** Xinbao Chen, Chenxi Wang, Chang Liu, Xiaodong Zhu, Yaohui Zhang, Tianxiang Luo, Junhao Zhang

**Affiliations:** 1 Sanya Research Institute, Hunan University of Science and Technology, Sanya 572025, China; 2 School of Earth Sciences and Spatial Information Engineering, Hunan University of Science and Technology, Xiangtan 411201, China; liuchang@mail.hnust.edu.cn (C.L.); zhuxiaodong2302@gmail.com (X.Z.); eminem@mail.hnust.edu.cn (Y.Z.); 2101080206@mail.hnust.edu.cn (T.L.); 2001080232@mail.hnust.edu.cn (J.Z.)

**Keywords:** pavement crack detection, UAV inspection system, SWM, MRC-YOLOv8, mountainous road

## Abstract

Road cracks significantly affect the serviceability and safety of roadways, especially in mountainous terrain. Traditional inspection methods, such as manual detection, are excessively time-consuming, labor-intensive, and inefficient. Additionally, multi-function detection vehicles equipped with diverse sensors are costly and unsuitable for mountainous roads, primarily because of the challenging terrain conditions characterized by frequent bends in the road. To address these challenges, this study proposes a customized Unmanned Aerial Vehicle (UAV) inspection system designed for automatic crack detection. This system focuses on enhancing autonomous capabilities in mountainous terrains by incorporating embedded algorithms for route planning, autonomous navigation, and automatic crack detection. The slide window method (SWM) is proposed to enhance the autonomous navigation of UAV flights by generating path planning on mountainous roads. This method compensates for GPS/IMU positioning errors, particularly in GPS-denied or GPS-drift scenarios. Moreover, the improved MRC-YOLOv8 algorithm is presented to conduct autonomous crack detection from UAV imagery in an on/offboard module. To validate the performance of our UAV inspection system, we conducted multiple experiments to evaluate its accuracy, robustness, and efficiency. The results of the experiments on automatic navigation demonstrate that our fusion method, in conjunction with SWM, effectively enables real-time route planning in GPS-denied mountainous terrains. The proposed system displays an average localization drift of 2.75% and a per-point local scanning error of 0.33 m over a distance of 1.5 km. Moreover, the experimental results on the road crack detection reveal that the MRC-YOLOv8 algorithm achieves an F1-Score of 87.4% and a mAP of 92.3%, thus surpassing other state-of-the-art models like YOLOv5s, YOLOv8n, and YOLOv9 by 1.2%, 1.3%, and 3.0% in terms of mAP, respectively. Furthermore, the parameters of the MRC-YOLOv8 algorithm indicate a volume reduction of 0.19(×10^6^) compared to the original YOLOv8 model, thus enhancing its lightweight nature. The UAV inspection system proposed in this study serves as a valuable tool and technological guidance for the routine inspection of mountainous roads.

## 1. Introduction

**Figure 1:**
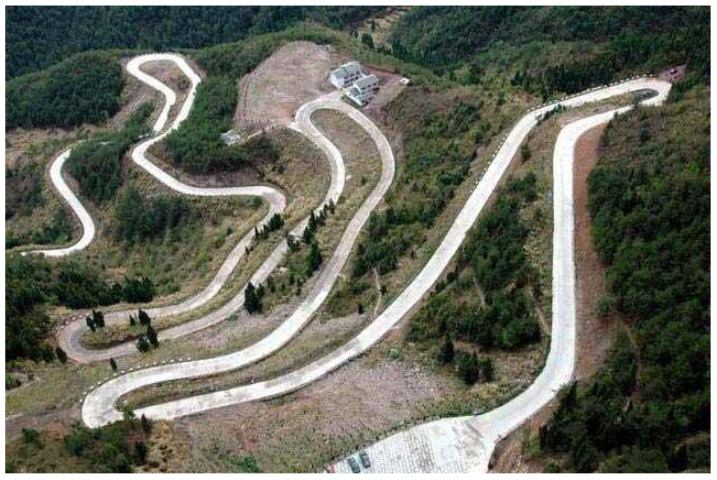
A mountainous road: the mountain road is narrow and winding (an example from a web resource [4]).

Road crack detection is crucial in monitoring the health of pavements to prevent the further deterioration and potential collapse of infrastructure [1]. With the increase in the number of vehicles and the continuous growth in road usage, urban roads, rural roads, mountain roads, and highways are experiencing varying degrees of deterioration. Meanwhile, road maintenance poses a significant challenge in the field of road transportation, particularly in China, where a large number of roads require routine maintenance. Cracks are one of the most challenging problems faced in road maintenance. Therefore, the timely detection and treatment of road defects can significantly reduce maintenance costs. Traditional methods, such as manual detection, are excessively time-consuming, labor-intensive, and inefficient. On the other hand, current multi-function detection vehicles equipped with various sensors are expensive and suitable for flat terrain such as urban roads or highways [2,3], but they are not widely available for mountain roads due to poor conditions, especially in very-high altitude or narrow areas. As shown in Figure 1, mountain roads typically present difficulties in ground access, steep and narrow characteristics, and bad roadbed hazards, which makes such inspection missions extremely difficult and time-consuming when using the existing manual inspection method or vehicle-mounted inspection.

In recent years, Unmanned Aerial Vehicles (UAVs) that carry a variety of micro-sensors have been widely applied in various inspection missions [5,6] due to their compact size, affordability, flexibility, and mobility. For instance, in the inspection of power transmissions, using an UAV inspection system instead of manual inspection can greatly reduce the risk of daily inspections [7]; meanwhile, UAVs have also gained increasing popularity in road detection and monitoring, which can greatly reduce the inspection time and extend to unknown complex environments. Liu et al. [8] proposed a systematic solution for automatic crack detection via UAV inspection with both hardware and software equipment, but the UAV lacked autonomous navigation and detection ability. In addition, UAVs are also gaining significance in the field of structural health performance inspections for civil engineering infrastructure, such as pipeline inspection [6,9] or bridge inspection [10]. Nevertheless, most existing UAV-based inspection systems [8,11,12] primarily focus on data acquisition, transmission, and some degree of pre-processing to achieve high-quality imagery. Moreover, inspectors still rely only on the visual inspection of images or partial computer vision methods to identify cracks [13]. However, the complexities of mountainous terrain and uneven roads pose challenges in manipulating flight routes and maintaining consistent vertical altitudes, thus making it difficult to obtain high-quality imagery. Additionally, the limited load capacity, computing ability, and endurance of small UAVs make it challenging to carry multiple sensors and to fly for extended periods [6]. For these reasons, existing commercial UAVs cannot be directly applied for this mission. Therefore, there is a need to design a high-performance, multi-functional, and reliable UAV inspection system with autonomous capabilities that is specifically tailored for mountainous roads and detecting defects such as pavement cracks.

Meanwhile, recent advancements in deep learning have had a profound impact on automating crack detection from road imagery [1,5,8,11,12,13,14]. These deep learning algorithms can be categorized into two major approaches, namely ‘two-stage’ algorithms and ‘one-stage’ algorithms. Each method has its own benefits; the single-phase algorithm is quicker, while the two-phase algorithm is more precise. Krizhevsky et al. [15] introduced a deep CNN architecture specifically for image classification, with a focus on detecting distress in asphalt pavements. Xu et al. [16] performed experiments to assess the effectiveness of Faster R-CNN and Mask R-CNN, and they compared their performance in various scenarios. Meftah [17] combined a random forest classifier with CNNs, integrating models like MobileNet, InceptionV3, and Xception and achieving an impressive validation accuracy of 99.97%. During the application of ‘one-stage’ algorithms, YOLO (You Only Look Once) serial algorithms are the most well known for fast one-stage object detection methods. Su et al. [18] proposed a YOLOv3-based solution that exhibited high precision, recall, and F1-Scores exceeding 95%. Zhou et al. [19] introduced YOLOv5s-attention, an enhanced version of the YOLOv5 algorithm that showcases superior accuracy, F1-Score, and mAP@0.5 compared to its predecessor. Jiang et al. [1] presented a self-attention road trap detection algorithm of RDD-YOLOv5 based on UAV detection, which can effectively detect road defects and has high practicability, achieving a mean average precision of 91.48%, thus surpassing the original YOLOv5 by 2.5%. Xiang X. et al. [12] proposed the GC-YOLOv5s model as a lightweight and easily deployable detector for UAV road crack detection, with a mAP@0.5 of 74.3%. Wang et al. [13] presented an improved BL-YOLOv8, which enhances the detection accuracy of road defects compared to the original YOLOv8 model, with an improvement of 3.3% in mAP@0.5 with 90.7%, and as well as a reduction of 29.92% in the parameter volume. The integration of deep learning has evidently significantly enhanced the efficiency, accuracy, and objectivity of road crack detection, thus opening avenues for further research in diverse environmental settings such as mountainous roads.

In particular, the YOLOv8 [20] algorithm, which offers significant improvements in both detection accuracy and speed, is the latest stability version of the YOLO series [21,22]. Additionally, YOLOv8 has a smaller weight file, being 90% smaller than YOLOv5. This makes it suitable for real-time detection on embedded devices. Compared to previous versions of the YOLO series, YOLOv8 excels in detection accuracy, lightweight characteristics, and fast detection time [22]. In the context of UAV-based pavement crack detection, being lightweight, accurate, and efficient are crucial characteristics due to limited computing and power ability. Therefore, we decided to choose the model using the latest stability YOLOv8s framework with further improvements, and we then compared the algorithm’s accuracy with other deep learning models.

Currently, road crack inspection technologies have unavoidably evolved from manual measurements to automatic inspections supported by computer technologies and deep learning algorithms. These automatic inspection methods are gaining popularity due to their efficiency and accuracy [5]. While current studies have mainly focused on urban roads, intercity highways, and flat rural roads using road inspection vehicles equipped with various sensors, there is a gap in the literature when it comes to crack detection on mountainous roads. UAVs can provide a flexible, simple, fast, and cost-effective solution for this challenge. While drones are predominantly utilized for capturing road crack image data [23,24,25], there is limited research on systems that integrate road automatic flight navigation with real-time crack detection. Furthermore, there is a lack of commercial UAVs specifically designed for crack detection on mountainous roads, thus hindering a potential area for future research and practical application. Therefore, this study aims to present a novel UAV inspection system designed for automatically detecting pavement cracks on mountainous roads. The UAV is equipped with vision and GPS/IMU sensors to autonomously follow a predefined path at a consistent altitude from the road pavement. The onboard computer processes images captured by the vision sensors to detect and recognize objects. Engineers can access real-time detection results and raw image data on an offboard computer.

The main contributions of our works can be summarized as follows: (i) An overall integrated architecture for UAV inspections system is proposed, including a UAV hardware system and software system, as well as advanced algorithms for autonomous crack detection, such as the improved YOLOv8. (ii) We developed a visual sliding window method to extract the flight routes along mountainous roads for the path planning and UAV autonomous navigation during GPS-denied or GPS-drift periods. Furthermore, we used the fusion strategy method to compare them with the GPS trajectories. (iii) A benchmark dataset of concrete and asphalt cracks from various roads was created and integrated, and this was then made publicly available for the research community, thus serving as a valuable supplement to existing crack databases.

This paper is structured as follows: Section 2 offers a comprehensive description of the primary hardware components of the UAV inspection system via detailing the components, and it also introduces the software architecture along with autonomous navigation and UAV data acquisition. Section 3 elucidates the autonomous crack detection method using the enhanced MRC-YOLOv8 algorithm. Section 4 delves into the experimental scenarios and results, while Section 5 engages in discussions on the experimental findings. Lastly, Section 6 encapsulates the key findings and conclusions of the research.

## 2. Architecture of the UAV Autonomous Inspection System

### 2.1. Hardware System

**Figure 2:**
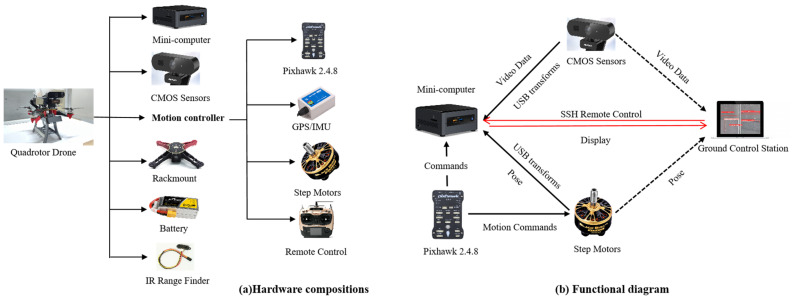
Hardware architecture of the UAV inspection system (modified from [9]).

To complete the inspection of pavement cracks on mountainous roads, a UAV must be equipped with a variety of sensors to ensure flight stability and to perform necessary tasks. The overall hardware architecture of our UAV inspection system is illustrated in Figure 2. The system consists of a mini-computer, two CMOS sensors, a motion control system, an Infrared Range Finder, and a rack mount. The UAV platform is supported by a folding carbon fiber rack with a Pixhawk autopilot. The motion controller, which includes a Pixhawk (v2.4.8), four step motors, a GPS/IMU unit, and a remote control, is responsible for guiding the UAV during autonomous flight. An IR Range Finder is utilized to measure the distances from the ground and to detect obstacles like trees or power lines. All sensors and controllers are connected to the mini-computer, which handles tasks such as processing pose data from the GPS/IMU, RGB video data from the mono-camera, and encoder data from the step motors. The processed results can be transmitted to a Wi-Fi-connected laptop for display and further analysis. The entire system is powered by a 14.8 V lithium battery. The key hardware components of the UAV inspection system are detailed in Table 1.

### 2.2. Software Systems

**Figure 3:**
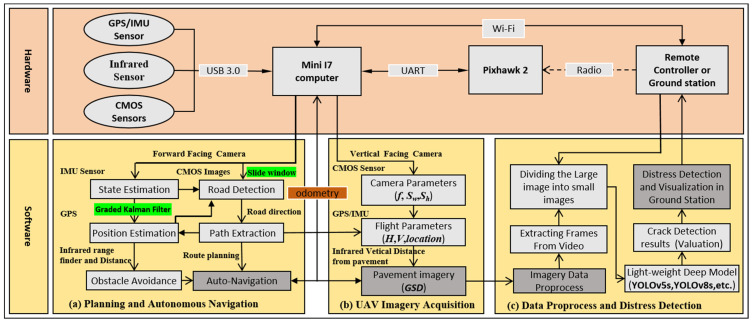
The overall framework of the UAV inspection system for pavement crack detection on mountainous roads.

In order to achieve a comprehensive integration of hardware, algorithms, and communication with various sensor devices, we utilized the open-source Robot Operating System (ROS, v1.0). The infrared data, GPS/IMU data, and mono-camera inputs were processed by a mini-computer to generate the source data stream. Additionally, the onboard computer was mounted on a custom-designed frame to capture inspection images, identify target objects from the camera feed, and process the GPS information from the flight controller on the UAV. The integrated hardware and software architectures for autonomous UAV crack inspections in mountainous road environments are depicted in Figure 3.

#### 2.2.1. Planning and Autonomous Navigation

(1) Flight Controller and Auto Vertical Height

**Figure 4:**
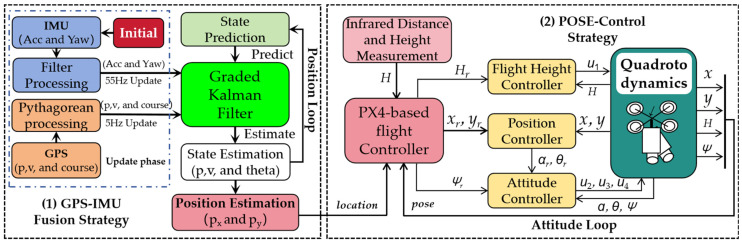
Cascaded control scheme of the quadrotor drones (modified from [26,27]).

In order to ensure the autonomous navigation and flight operations of UAVs along mountain roads and terrains, it is crucial to maintain autonomous flight and a constant altitude for the UAVs. This can be accomplished through two primary strategies (which are depicted in Figure 4): (1) the GPS/IMU fusion strategy for autonomous flight, and (2) the POSE Control Strategy to ensure the drone’s attitude and altitude. Initially, we utilized GPS/IMU for the Pixhawk 2 position estimation and TSDF’s 6 DOF for pose estimation as the input. A fusion strategy known as the graded Kalman filter [26] was then used to integrate the IMU and GPS positioning information in order to estimate the current position and state of the UAV. Additionally, a cascade control loop was employed for control, addressing both the inner loop control (attitude loop) and the outer loop control (position loop). Among the control methods investigated, the composite nonlinear feedback (CNF) method for attitude stabilization and the robust perfect tracking (RPT) method [27] for trajectory tracking have demonstrated superior performances. The flight control laws were implemented using a PX4-based flight controller in this study.

The graded Kalman filter, which closely follows the steps of the traditional Kalman filter in its calculation process, was introduced in this study. This IMU state estimation model was utilized to estimate position and velocity values in the absence of GPS updates from the sensor. The state was updated using IMU inputs as described below [26]:

(1) $${\overset{\vee }{X}}_{k}=FX_{k-1}+Bu=\left[\begin{array}{cc} 1 & \Delta t\\ 0 & 1 \end{array}\right]X_{k-1}+\left[\begin{array}{c} \Delta t^{2}\\ \Delta t \end{array}\right]u,$$

where ${\overset{\vee }{X}}_{k}=\left[\begin{array}{c} \overset{\vee }{p_{k}}\\ \overset{\vee }{v_{k}} \end{array}\right]$, $u=\left[a\right]$, $\overset{\vee }{P_{k}}$ is the distance-traveled prediction, $\overset{\vee }{v_{k}}$ is the velocity prediction, and a is the magnitude of acceleration.

When a GPS position is available and there is an update from the GPS, the graded Kalman filter will utilize the GPS estimation model described below (where it first fuses with the GPS velocity) as follows:

(2) $$K_{v|k}={\overset{\vee }{P}}_{v|k-1}{\left({\overset{\vee }{P}}_{v|k-1}+Q_{k}\right)}^{-1},$$

(3) $${\overset{\vee }{v}}_{k}={\overset{\vee }{v}}_{k-1}+K_{k}(U_{k}-{\overset{\vee }{v}}_{k-1}),$$

(4) $${\overset{\vee }{P}}_{v/k}=Q_{k}-K_{v|k}Q_{k},$$

where $K_{v|k}$ is the Kalman gain from velocity, $\overset{\vee }{v_{k}}$ is the estimated velocity, and ${\overset{\vee }{P}}_{v/k}$ is the estimated velocity error covariance. Secondly, it fuses with the GPS distance value as follows:

(5) $$K_{p/k}={\overset{\vee }{P}}_{p|k-1}{\left({\overset{\vee }{P}}_{p|k-1}+S_{k}\right)}^{-1},$$

(6) $${\overset{\vee }{p}}_{k}={\overset{\vee }{p}}_{k-1}+K_{k}(V_{k}-{\overset{\vee }{p}}_{k-1}),$$

(7) $${\overset{\vee }{P}}_{p/k}=S_{k}-K_{p|k}S_{k},$$

where $K_{p|k}$ is the Kalman gain from the traveling distance, $\overset{\vee }{p_{k}}$ is the estimated velocity, and ${\overset{\vee }{P}}_{p/k}$ is the estimated velocity error covariance.

(2) Path Extraction and Autonomous Navigation

In this study, the GPS/IMU based sensor fusion can be directly employed for state estimation and also for the autonomous flying of UAVs, which demonstrate its accuracy performance. However, it is known to suffer from inaccuracies and drift in the GPS signal. This poses a challenge in areas with compromised GPS reception, particularly in our experimental region covered with forests or valleys surrounded by mountains [28,29]. To tackle this issue, a novel approach is proposed for localizing UAVs and enabling route navigation in mountainous terrains with limited GNSS coverage. The proposed approach involves utilizing the visual sliding window algorithm to calculate the angle and travel distance between two consecutive captured images. Furthermore, we utilized the altitude of the UAV, which is determined by the integrated barometer sensor in the autopilot, to accurately measure the actual distance using the ground sample distance (**GSD**) method.

**Figure 5:**
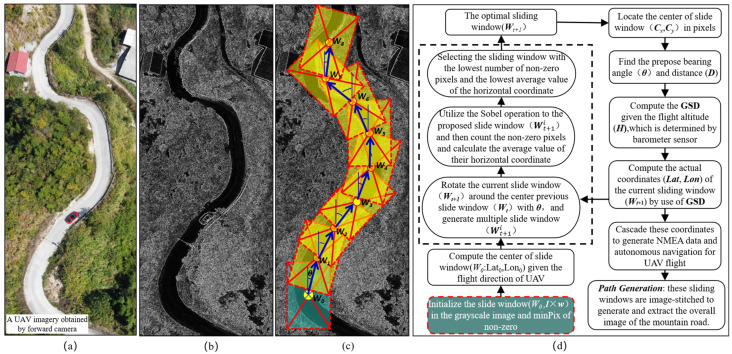
The workflow of the sliding window method for route generation: (**a**) RGB image; (**b**) grayscale image; (**c**) route generation with SWM in the grayscale image; and (**d**) the workflow of the slide window method (SWM).

The algorithm workflow of the visual sliding window method is depicted in Figure 5. Initially, the RGB image (Figure 5a) from the UAV imagery is converted to a grayscale image (Figure 5b). The distinct brightness contrast between the road and the surrounding vegetation aids in clearly distinguishing them. In the grayscale image (Figure 5b), the road appears as a consistent pixel pattern, facilitating the identification of its outline and direction. Therefore, grayscale value maps are preferable for the autonomous capture, tracking, and navigation of mountain roads and other aerial tasks during UAV aerial photography.

Based on the grayscale image (Figure 5c), we interpreted the process of route generation with the SWM method. Firstly, parameters such as the size of the initial sliding window (***l*** × ***w***) and the minimum number of non-zero pixels in the sliding window (minPix) were set. The initial sliding window should encompass the entire width of the road, with the length of the sliding window (***l***) indicating the direction of travel, and the center of the sliding window serving as the initial position. Subsequently, multiple proposed sliding windows ($W_{t+1}^{i}$) were generated by rotating (***θ***) the specific sliding window ($W_{t+1}^{0}$) around the center point of the previous sliding window (***W****_t_*), thus acting as the axis. The Sobel operation was then applied to these specific sliding windows to extract the road features, and this was followed by counting the number of non-zero pixels and calculating the average value of the horizontal coordinates of all non-zero pixels. The optimal sliding window was determined by selecting the sliding window with the lowest number of non-zero pixels and the lowest average value of the horizontal coordinates. Furthermore, the actual latitude and longitude coordinates (***Lat***, ***Lon***) of the current sliding window (***W***_t+1_) were computed based on the central pixel coordinates of the optimal sliding window, the rotation angle (***θ***), the offset (***D***) of the adjacent sliding window, the ground sampling distance (**GSD**) of the known camera, and the current altitude (***H***) of the UAV. The altitude (***H***) was determined by the barometer sensor embedded in the autopilot. Subsequently, following the completion of the sliding window detection, GNSS-coordinate data files in the NMEA format are generated for route planning of the mountain roads and the autonomous navigation of the UAV. The autopilot is equipped with a port to receive NMEA data from the GPS receiver. Hence, the computed coordinates are converted to the standard NMEA format and fed to the autopilot through its GPS input port [29]. Finally, all connected sliding windows are superimposed with the original images to extract the entire mountain road from UAV imagery.

#### 2.2.2. UAV Data Acquisition for Mountainous Roads

**Figure 6:**
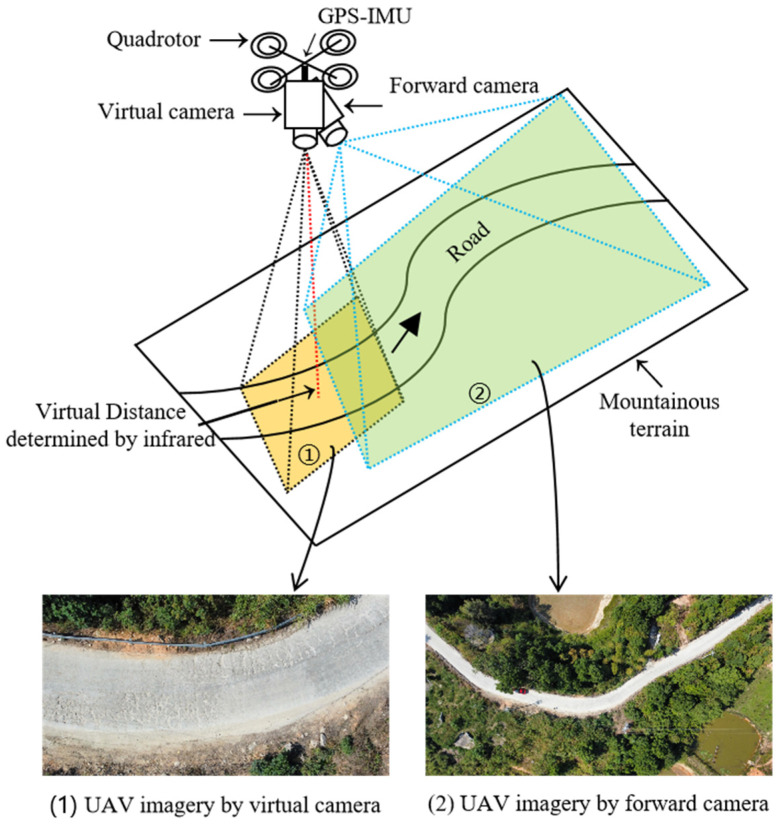
A diagram of the UAV data acquisition process.

The acquisition of data imagery in our UAV inspection system was divided into two parts: local data imagery for road pavements and global data imagery for mountainous terrains. Figure 6 illustrates the diagram of the UAV data acquisition process. The forward camera is mainly used to capture the global mountainous terrain, thus assisting the drone in tracking ground road feature points and landmarks for autonomous positioning and navigation. It is a compact camera that provides a comprehensive view of the scene. On the other hand, the vertical camera is a high-definition camera that focuses on the local field of view scene and is primarily used for identifying and detecting road crack targets. Additionally, to determine the optimal altitude of the UAV and ensure efficient flight, the following considerations should be taken into account: (i) the UAV camera view should cover the full width of the road that needs to be inspected; (ii) it is important to avoid any interference from auxiliary facilities such as roads, trees, and street lights during the flight; and (iii) it is crucial to maintain a constant vertical distance from pavements, maintain a consistent speed, and capture vertical imagery to minimize image distortion.

## 3. Pavement Autonomous Crack Detection with Improved YOLOv8

### 3.1. Dataset Collection and Labeling

Our dataset, which was utilized in this experiment, comes partially from self-made datasets and partially from public concrete cracks dataset [30]. The road cracks mainly come from concrete paved roads. The self-made dataset was collected using a Huawei cellphone and DJI M3 equipped with a 1/2.3 CMOS camera with a resolution of 12 megapixels. The images were taken at a low height range of 1~3 m, resulting in a total of 658 road crack photos with a resolution of 3840 × 2160. Various real-world road environments were selected for photography, including rural and mountainous roads, which are commonly surfaced with concrete materials in China. As shown in Table 2, the photos captured a diverse range of crack morphologies in concrete pavements, including Horizontal Cracks (HCs), Vertical Cracks (VCs), Oblique Cracks (OCs), Pothole cracks (PCs), Block Cracks (BCs),and Expansion Joints (EJs). It is important to note that “expansion” joints are incorporated in concrete to prevent the expansive cracks caused by temperature changes, which are not classified as crack types with damage.

To optimize the parallel batch computing speed, deep learning models require specific requirements for training image datasets. For instance, images are often cropped to 640 × 640 or 320 × 320 pixels to improve model efficiency during training. The self-custom original images extracted from UAV imagery should be cropped, resized, and standardized to ensure consistent specifications. In this experiment, extracted frame images were used as the initial dataset and cropped into 640 × 640 pixel images. Data enhancement techniques such as augmentation, translation, flipping, and rotation were employed to increase the number of samples for crack categories. The labeling tool was utilized to decode, annotate, and categorize various types of cracks in the UAV imagery cracks dataset from mountainous roads. In this study, the dataset consisted of a total of 5220 images, including 1888 HC cases, 1548 VC cases, 1565 OC cases, 1590 BC cases, 316 PC cases, and 300 EJ cases. The dataset was divided into training, validation, and test sets, with ratios of 80%, 10%, and 10%, respectively, to evaluate the effectiveness of the deep learning model.

### 3.2. Detection Method Based on the Enhanced YOLOv8 Model

The YOLOv8 model is a state-of-the-art object detection model that incorporates three scale-detection layers to address the multiscale nature of objects. However, when applied to crack detection using image data, challenges arise due to complex backgrounds and the heavy workload required for crack identification. The existing detection structure of YOLOv8 is insufficient in meeting the demands for efficient and accurate crack identification. To overcome these challenges, we propose enhancements to the YOLOv8 model, including an improved loss function, attention mechanism, and multiscale feature fusion. These enhancements aim to optimize the YOLOv8 model for rapid and precise crack recognition. Specifically, we introduce the Efficient IOU Loss Function (EIOU) [31], which is based on the Complete Intersection over Union (CIOU) [32], to handle the varying aspect ratios of the bounding boxes (BboxLoss), as well as the Dilation-Wise Residual (DWR) module [33] to enhance selectivity in complex backgrounds. These improvements are designed to address the current limitations of the YOLOv8 model and enhance its performance in crack detection scenarios. Additionally, we developed a crack detection method named MRC-YOLOv8 (YOLOv8 for Mountainous Road Cracks Detector), which balances lightweight implementation and algorithmic performance.

**Figure 7:**
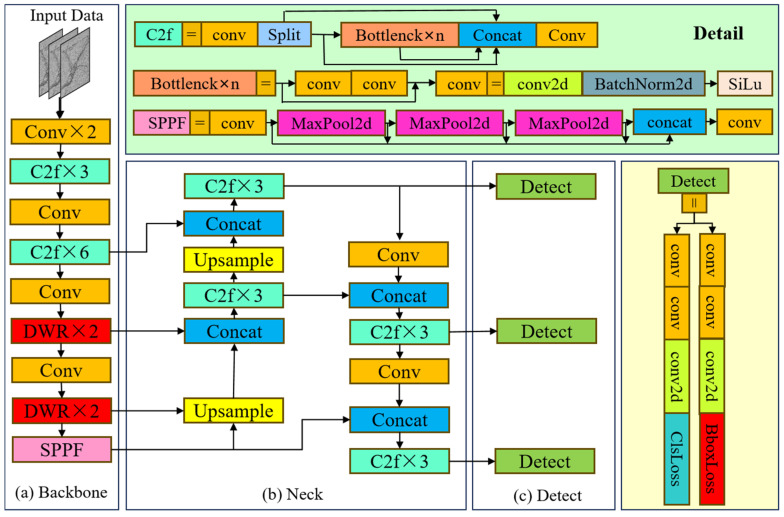
The basic network and some improvements (marked in red rectangles) of the enhanced YOLOv8 structure.

Figure 7 illustrates the basic structures and some improvements (marked in the red rectangles) of our enhanced YOLOv8 algorithm, which includes an input section, as well as a Backbone, a Neck, and a Detect part in the output segment. The input section performs mosaic data augmentation, adaptive anchor computation, and adaptive gray padding on the input image. The Backbone and Neck networks are crucial components in the YOLOv8 architecture. The input image is processed through various Conv and C2f modules to generate feature maps at different scales. The C2f module is an upgraded version of the original C3 module and serves as the primary residual learning component. By incorporating the ELAN structure from YOLOv7 [34], it enhances the gradient flow by eliminating a standard convolutional layer and maximizes the use of the Bottleneck module. This approach maintains a lightweight design while capturing a broader range of gradient information.

#### 3.2.1. EIOU Function

To address the issue of varying aspect ratios, the MRC-YOLOv8 model utilizes the CIOU as the bounding box regression function. CIOU is a metric used in target detection tasks to assess the similarity between bounding boxes. Unlike the traditional IOU (Intersection over Union) measure, CIOU takes into account not only the position, but also the size and aspect ratio of the box. This is crucial because IOU may not accurately represent box similarity as it focuses only on position. By factoring in the center-point distance, aspect ratio difference, and box size in its calculation, CIOU provides a more comprehensive evaluation of box similarity. When considering a predicted Box B and a target Box $B^{\mathrm{gt}}$, with the central points denoted as *b* and $b^{\mathrm{gt}}$, respectively, the CIOU loss function is designed to encompass these factors for enhanced accuracy in similarity measurements. The CIOU loss function [32] is defined as follows.

(8) $$L_{\mathrm{CIOU}}=1-\mathrm{IOU}+\frac{\rho^{2}\left(b,b^{\mathrm{gt}}\right)}{c^{2}}+\alpha v\;,$$

(9) $$\alpha =\frac{v}{\left(1-\mathrm{IOU}\right)+v},$$

(10) $$v=\frac{4}{\pi^{2}}{\left(\arctan\frac{w^{\mathrm{gt}}}{h^{\mathrm{gt}}}-\arctan\frac{w}{h}\right)}^{2},$$

where $\rho \left(\cdot \right)=∥b-b^{\mathrm{gt}}{∥}^{2}$ indicates the Euclidean distance, *c* is the diagonal length of the smallest enclosing box covering the two boxes, and the gradients of *v*, *w*, *r*, *t*, *w*, and *h* are calculated as follows.

(11) $$\frac{\partial v}{\partial w}=\frac{8}{\pi^{2}}\left(\arctan\frac{w^{\mathrm{gt}}}{h^{\mathrm{gt}}}-\arctan\frac{w}{h}\right)\times \frac{h}{w^{2}+h^{2}},$$

(12) $$\frac{\partial v}{\partial h}=-\frac{8}{\pi^{2}}\left(\arctan\frac{w^{\mathrm{gt}}}{h^{\mathrm{gt}}}-\arctan\frac{w}{h}\right)\times \frac{h}{w^{2}+h^{2}}.$$

However, since *v* only reflects the discrepancy of the aspect ratio, rather than the real relationship between the predicted boxes of the real box, the CIOU loss may optimize the similarity in an unreasonable way. To address this problem, we changed the CIOU loss to a more efficient version of IOU loss, i.e., EIOU loss [31], which is defined as follows:

(13) $$L_{\mathrm{EIOU}}=L_{\mathrm{IOU}}+L_{\mathrm{DIS}}+L_{\mathrm{ASP}},$$

(14) $$L_{\mathrm{EIOU}}=1-\mathrm{IOU}+\frac{\rho^{2}\left(b,b^{\mathrm{gt}}\right)}{{\left(w^{c}\right)}^{2}+{\left(h^{c}\right)}^{2}}+\frac{\rho^{2}\left(w,w^{\mathrm{gt}}\right)}{{\left(w^{c}\right)}^{2}}+\frac{\rho^{2}\left(h,h^{\mathrm{gt}}\right)}{{\left(h^{c}\right)}^{2}}.$$

Here, the loss function consists of three parts: the IOU loss *L*_IOU_, the distance loss $L_{\mathrm{DIS}}$, and the aspect loss $L_{\mathrm{ASP}}$ (i.e., the overlapping area, center-point example, and aspect ratio, respectively). The height-width loss specifically aims to reduce the differences in height and width between the predicted target bounding box and the actual bounding box, leading to quicker convergence and enhanced localization accuracy.

#### 3.2.2. DWR Segmentation

**Figure 8:**
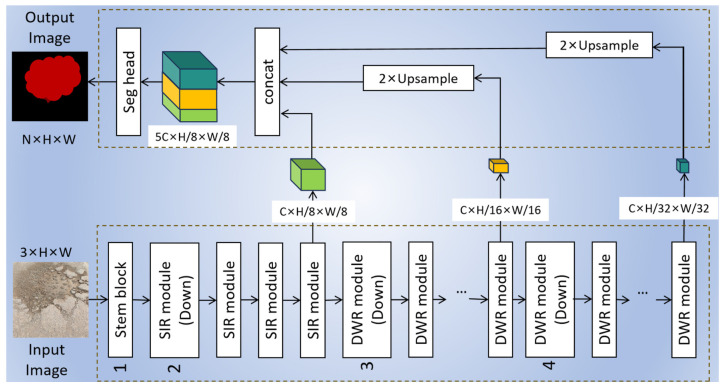
DWR segmentation model structures (modified from [33]).

In the MRC-YOLOv8 model, some original C2f modules in the original YOLOv8 were replaced with DWR modules to simplify the model and reduce parameter count. The DWRSeg network incorporates an attention mechanism, utilizing a novel Dilation-Wise Residual (DWR) module and a Simple Inverted Residual (SIR) module at both high and low levels. The network architecture, illustrated in Figure 8, is known for its simplicity and efficiency. The attention mechanism in the DWRSeg network enhances important image information by prioritizing specific areas, thus facilitating information fusion and interaction across different levels or scales. This selective amplification of feature channels containing target information enhances the model’s performance. The network structure comprises a DWR module and an SIR module for high- and low-level features. The encoder–decoder configuration includes four stages: the stem block, the low stage of SIR modules, and the two high stages of DWR modules. The DWR module captures multiscale contextual information and efficiently fuses feature maps, while the SIR module is tailored for smaller receptive field sizes in the lower stage to enhance feature extraction efficiency.

### 3.3. Training Process of the Improved YOLOv8 Model

This study utilized various deep learning algorithms in a consistent configuration, as outlined in Table 3. To compare the MRC-YOLOv8 performance, pretrained models like YOLOv5s, the original YOLOv8n, and the latest YOLOv9n were employed, with code implementation conducted in Python using the Pytorch framework. The experiments ran on a computer with an Intel Xeon(R) Platinum 8352 V processor and 64 GB memory. Hyperparameters were set with a unified input image size of 640 px × 640 px, 200 training iterations, a batch size of 16, and a learning rate of 0.001. The learning rate decay followed the Cosine Annealing algorithm, with the optimizer being SGD and the Momentum set to 0.937.

## 4. Experiments and Results

### 4.1. Experimental Scenarios

**Figure 9:**
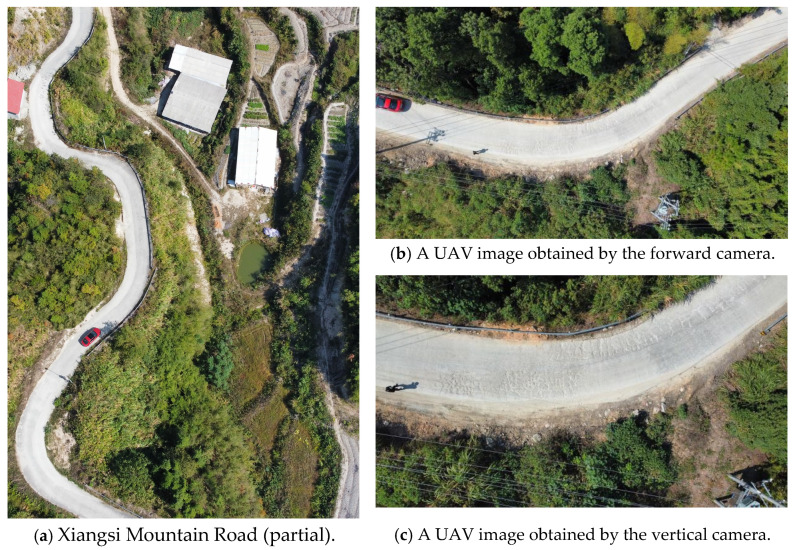
Xiangsi Mountainous Road in this study.

In this experiment, the flight mission was conducted on Xiangsi Mountain Road in Yueyang County, Hunan Province, China. Xiangsi Mountain Road is a concrete pavement with a width of 4 m. The road reaches a maximum height of 957.8 m above sea level and a minimum of 785.2 m, resulting in a height difference of 172.6 m. The road features a maximum bend of 45 degrees. The UAV aerial photography covered a distance of 2.4 km during the experiment. Constructed and opened to traffic in 2015, the road has shown visible damage over the past 7 years, including transverse cracks, longitudinal cracks, and alligator cracks. The experiment took place on 10 October 2023 at 10:00 am under sunny conditions with light traffic. Utilizing UAV flight parameters and relevant formulas, the initial flight height (***H***) was set at 21.5 m, and the initial flight velocity (***v***) was set at 3.5 m/s. The forward camera captured imagery of the mountainous road from a global perspective, while the vertical camera captured imagery of the pavement. Figure 9 displays the partial imagery results of Xiangsi Mountain Road.

### 4.2. Experimental Results

#### 4.2.1. Results for Autonomous Navigation Using UAV Forward Imagery

(1) Three strategies of visual windows for route generation

**Figure 10:**
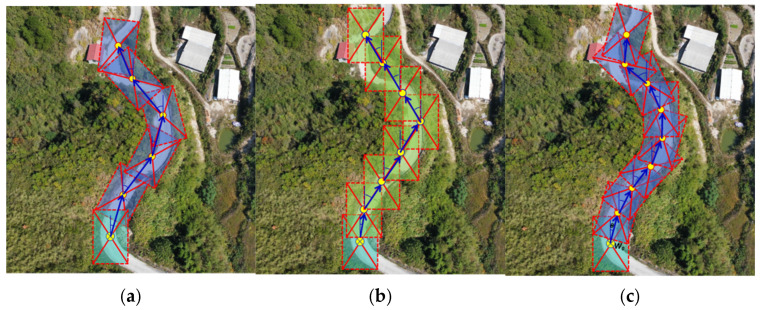
The three strategies of the sliding window method: (**a**) the first strategy; (**b**) the second strategy; and (**c**) the third strategy.

In order to accurately extract the flight route of a UAV on a mountainous road, this study conducted several implementations of route generation from the UAV imagery obtained by a forward camera. Figure 10 illustrates the three strategies of the sliding window method that were used to demonstrate the overall process of route generation. (a) The first strategy involves adjacent sliding windows with a forward superposition degree of 20~30%. The forward sliding window rotates at an arbitrary angle to extract the road through frame selection. This strategy has the fastest implementation algorithm and achieves more accurate road extraction. However, the generated route does not align well with the road curvature. (b) In the second strategy, the adjacent sliding windows have a forward superposition degree of 50%, and the rotation is directly canceled. Instead, the forward sliding window is used for lateral translation to select and extract the road. This strategy has a slower implementation algorithm and poor road extraction effect, resulting in an incomplete extracted road. Additionally, the generated route had the worst fit with the road curvature. (c) In the third strategy, the adjacent sliding windows have a forward superposition degree of 50%, and the rotation angle of the forward sliding window is arbitrary. The road is extracted through frame selection. Although the implementation algorithm of this strategy runs slowly, it achieves optimal road extraction with a complete extracted road. Moreover, the generated route exhibits the highest consistency with the road curvature. Therefore, we utilized the third strategy in this experiment to generate and extract road routes.

(2) Comparison of the route generation results

**Figure 11:**
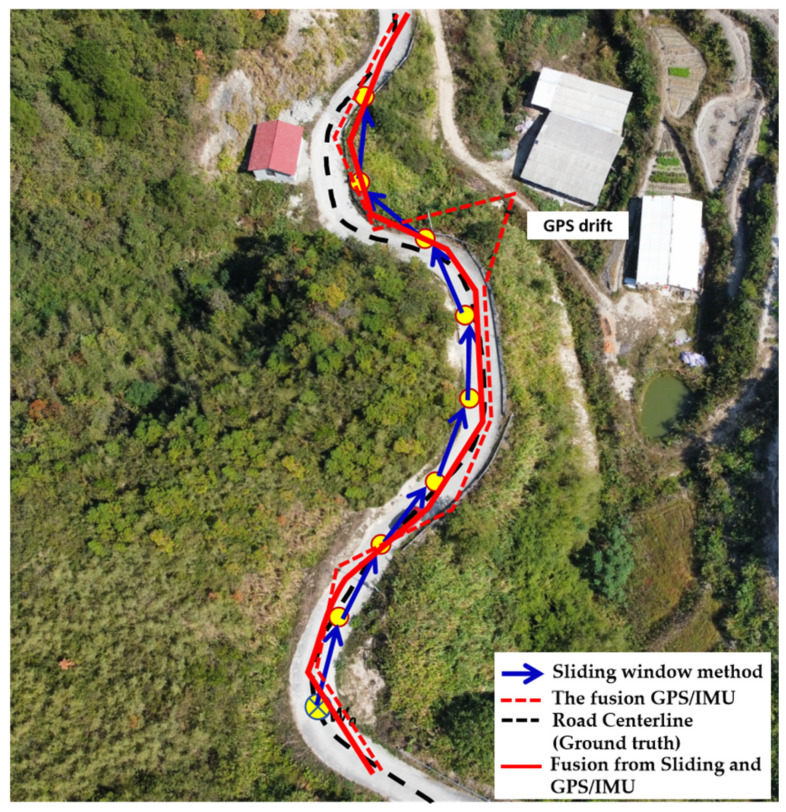
A comparison of the route generation results from the experiment.

To evaluate the effectiveness of our proposed method, this study conducted a comparative analysis of the route generation using the customized UAV inspection system. The experimental scenario involved simulated data, which were analyzed with the graded Kalman filter [26]. Figure 11 illustrates a comparison of the route generation results, highlighting the potential of the proposed sliding window method to compensate for GPS/IMU positioning errors and to even replace GPS/IMU data, particularly in GPS-denied or GPS-drift mountainous roads during UAV flights. In this figure, the fusion GPS positions (represented by the red dotted line) obtained from the GPS/IMU sensor were plotted for each second of received information. The simulated positioning was also calculated using the proposed sliding window method, and the results were shown through the blue arrow line in Figure 11. A general comparison with the ground truth (represented by the black dotted line) revealed that both routes experienced some positioning errors, especially the GPS/IMU sensor that had a max. 4 m visible error GPS drift. Furthermore, a fusion method was employed to integrate the GPS/IMU data and the simulated data. The results demonstrate that the fusion route closely aligns with the road centerline, specifically the ground truth.

We also calculated the error using the Root Mean Squared Error (RMSE) method to obtain an exact number on how much error was generated from each method compared to the benchmark. We performed this by comparing the positioning results from the sliding window method, the fusion of GPS/IMU, and the fused trajectory with the ground truth. The RMSE formula is given as follows:

(15) $$RMSE=\sqrt{\frac{1}{m}\sum_{i=1}^{m}{\left(y_{i}-\overset{\wedge }{y_{i}}\right)}^{2}}.$$

Table 4 presents a comparison of the RMSE values among the three methods for an experimental flight distance of 1.5 km. The results indicate that the SWM shows better GPS accuracy compared to the X and Y axes positions, albeit one that is slightly lower than the GPS/IMU model. The fusion strategy primarily involved the graded Kalman filter in this experiment. The fusion of GPS/IMU outperformed the standalone GPS model in terms of accuracy, with slightly better RMSE values for sensor positioning. The Y axis exhibited higher RMSE values than the X axis due to the predominant flight direction movements along the Y axis. In terms of the H axis, GPS/IMU (RMSE 0.323) performed better than GPS alone (RMSE 0.571). Despite GPS/IMU offering better accuracy in the total distance calculations, the final fusion method, which also utilized the graded Kalman filter, surpassed their performance with an RMSE value of 0.339, which is an improvement over the GPS/IMU’s RMSE of 0.427. Overall, the final fusion results demonstrated superior accuracy in the X axis, Y axis, and H elevation.

#### 4.2.2. Results for Autonomous Crack Detection Using UAV Vertical Imagery

(1) The running performance of the autonomous crack detection

To evaluate the performance of the lightweight, improved MRC-YOLOv8, this study conducted a comparison with the YOLOv5s model, as well as the original YOLOv8n and YOLOv9n (the latest evolution of the YOLO series) algorithms. The operational performance of the three algorithms is summarized in Table 5. The YOLO series models, known for being single-stage algorithms, exhibit notably faster running performances. Among them, the MRC-YOLOv8 model stands out with the highest number of parameters (2.81 × 10^6^) and minimal memory requirements (11.4 MB), while achieving superior frame rates (152.73 f·s^−1^) and training speed (1.12 h) compared to the other three YOLO series algorithms. When examining the same datasets, it was evident that our improved YOLOv8 model offers a superior running performance and requires specific environment configurations. The MRC-YOLOv8 algorithms are better suited for lightweight deployment in real-time detection tasks on UAV platforms, while the original YOLOv8 model performs equally well.

(2) The identification accuracy of the autonomous crack detection

To demonstrate the algorithms’ effectiveness for various models, the following four commonly used evaluation metrics were used in this study to access the identification accuracy: Precision (***P***), Recall (***R***), ***F***1-Score, Average Precision (***AP***), and Mean Average Precision (**mAP**). The results in Table 6 indicate that the MRC-YOLOv8 model also exhibited the highest accuracy compared to the other algorithms, thus surpassing the performance of the remaining YOLO models. Specifically, our improved YOLOv8 achieved values of 90.7% for Precision, 89.8% for Recall, 87.4% for F1-Score, and 92.3% for mAP. Notably, the latest iteration of YOLOv9n did not demonstrated optimal performance, exhibiting the lowest precision across the various metrics. It is evident that the current open-source version of YOLOv9 requires additional refinement.

**Figure 12:**
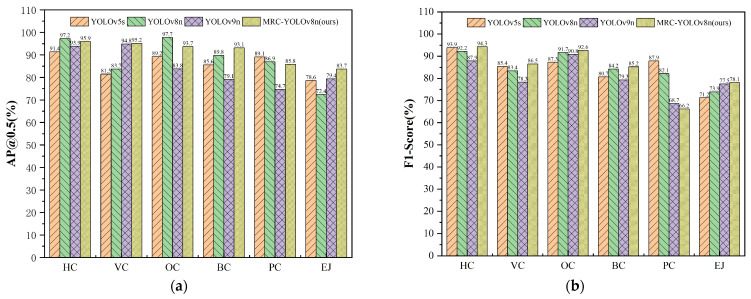
A comparison of the identification accuracy results of the three algorithms for the seven types of crack damage: (**a**) mAP@0.5 (%) and (**b**) F1-Score (%).

**Figure 13:**
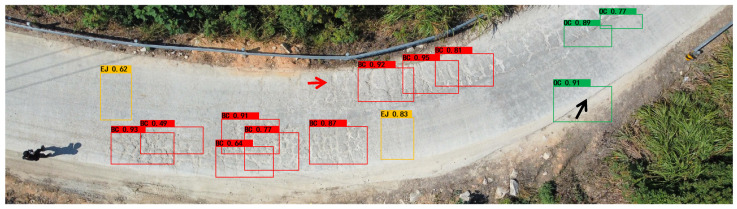
Partial visual results of the crack detection, based on MRC-YOLOv8, of the concrete pavements in our UAV vertical imagery in this experiment.

A comparative analysis was further conducted to highlight the discrepancies in the model recognition accuracy across different crack categories. Six types were considered: Horizontal Cracks (HCs), Vertical Cracks (VCs), Oblique Cracks (OCs), Block Cracks (BCs), Pothole Cracks (PCs), and Expansion Joints (EJs). The findings presented in Table 7 and Figure 12 demonstrate that the MRC-YOLOv8 algorithm outperformed the other three algorithms in various categories. Specifically, it achieved higher scores in VCs (mAP 95.2% and F1 86.5%), BCs (mAP 93.1% and F1 85.2%), PCs (mAP 85.8% and F1 66.2%), and EJs (mAP 83.7% and F1 78.1%). Accordingly, the MRC-YOLOv8 algorithm ranked second in HCs (mAP 95.9% and F1 94.3%) and OCs (mAP 93.7% and F1 92.6%). It is important to highlight that EJs exhibited the lowest accuracy, potentially due to confusion with the HC or VC classifications (please refer to the black arrow marker in Figure 13).

(3) The visual detection results based on improved YOLOv8

By comprehensively comparing the YOLO series algorithms, our proposed MRC-YOLOv8 outperformed the other YOLO series algorithms in both detection accuracy and speed. In this study, we further present the partial visual results of the crack detection on concrete pavements using the improved YOLOv8 model from the UAV vertical imagery captured in this experiment, as illustrated in Figure 13. The MRC-YOLOv8 model showed a better performance in identifying various crack types in the UAV imagery of mountainous roads. It is worth noting that the MRC-YOLOv8 model successfully identified the majority of BC types and their locations, while a few were only partially identified (indicated by the red arrow marker in Figure 13). This discrepancy may be attributed to variations in the crack scales.

## 5. Discussion

Mountainous roads are vulnerable to harm caused by natural calamities, significant temperature variances, substantial traffic, and rough topography. Pavement deterioration is prominently displayed through the presence of cracks, marking the initial phase of distress [35]. Hence, ensuring the periodical and precise identification of cracks is paramount for upholding road safety and averting additional harm. However, conventional inspection techniques and tools, like manual detection and multipurpose vehicles, are arduous, time-consuming, and ineffective. Furthermore, multipurpose detection vehicles featuring diverse sensors are costly and impractical for mountainous roads due to unfavorable road conditions.

To address these challenges, this study proposes a low-cost, customized UAV inspection system with high autonomous capabilities that are specifically designed for mountainous terrain. The proposed system integrates autonomous navigation methods and automatic pavement crack detection based on a deep learning model. It consists of an onboard mini-computer, a perception module, a path planning module, an acquisition module, and an on/offboard detection module. The perception module incorporates two monocular cameras, a GPS/IMU, and an Infrared Range Finder to serve as the basic sensor suite. Its primary function is to obtain target information and transmit processed data to the path planning module. The path planning module generates a flight path based on a pre-set GPS/IMU trajectory along mountainous roads (even in GPS-drift or GPS-denied environments) using the sliding window method of the UAV forward camera. During the autonomous flight, the acquisition module captures footage or takes photos of the pavement using the UAV’s vertical camera. Finally, during the detection phase, the on/offboard module generates a real-time spatial trajectory and visual results of the crack detection based on the improved YOLOv8 model. Table 8 provides a comparative analysis of the related research on these methods from various perspectives.

The proposed system also has the capability to store geographical information, including mountain road directions, and it can extract GPS trajectory information for the preliminary autonomous path planning of UAVs. This is a common approach used for autonomous drones in commercial applications. However, relying solely on geographic information (GIS) to plan the path of UAVs on mountainous roads presents several key challenges that hinder true autonomous navigation. The main challenges are as follows: (i) GIS provides planar geographic information, which is suitable for vehicle navigation but insufficient for drones that require three-dimensional spatial information. (ii) The drone’s flying area should maintain a safe distance from the road and consider factors such as wind direction. Thus, it requires a special attention toward safety. (iii) When the drone flies autonomously, it needs to avoid trees, which cannot be reflected in the GIS and requires on-site measurements. To address these challenges, this study also explored the potential solution of utilizing infrared scanning technology and our proposed sliding window method to aid in automatic planning and navigation.

The sliding window method can be effective in the context of mountainous roads that further detail are needed on. Mountainous roads have unique geographical characteristics, such as being single, narrow, curved, and steep, and they are often surrounded by forest vegetation and rocks. This makes the appearance and structure of mountain roads significantly different from the surrounding environment and vegetation. Computer vision techniques can utilize these features to identify, locate, and extract mountain roads from UAV imagery. To achieve the autonomous positioning and navigation of a UAV, the visual sliding window method is employed to detect the road direction based on the color and zigzag structure of the mountain road. This study explored three strategies for generating and extracting routes when using the sliding window method for UAV autonomous navigation along a desired road. The experimental results demonstrate that our fusion method can perform real-time route planning in GPS-denied mountainous environments, with an average localization drift of 2.75% and a per-point local scanning error of 0.33 m over a traveling distance of 1.5 km.

Meanwhile, the MRC-YOLOv8 model is proposed for utilization in UAV autonomous crack detection scenarios on mountainous roads. The original YOLOv8 enhances the model structure and training strategy of YOLOv7 to boost detection speed and accuracy. For instance, the incorporation of Extended-ELAN (E-ELAN) as a long-range attention network improves feature extraction capabilities. Additional data augmentation techniques like Mosaic + MixUp are employed to bolster generalization and robustness. Nevertheless, mountain road crack detection heavily relies on the quality of drone aerial images, which can be affected by factors like roadside tree shadows and road unevenness. To address these challenges, we enhanced the model and upgraded the YOLOv8 algorithm. These enhancements involve the integration of a penalty term named EIOU, which is derived from CIOU, to effectively handle the diverse aspect ratios of the bounding boxes in remote sensing images. Additionally, we introduced the Dynamic Weight Redistribution (DWR) module to improve the model’s ability to discern details in complex image backgrounds by dynamically adjusting the convolution kernel weights based on input data characteristics. The experimental results indicate that our MRC-YOLOv8 algorithm achieves higher recognition accuracy compared to YOLOv5s, YOLOv8n, and the latest YOLOv9n algorithms, surpassing them by 1.2%, 1.3%, and 3.0% in terms of mAP, respectively. Additionally, the parameters of the MRC-YOLOv8 algorithm show a reduction of 0.19 (×10^6^) in volume compared to the original YOLOv8 model, thus making it more lightweight. Moreover, the detection speed increased by 12.05 FPS (frames per second), thus making it faster. In summary, MRC-YOLOv8 is a lightweight, automatic, and real-time crack-detecting method that can be integrated into a UAV inspection system, thus significantly reducing the human resources required for regular distress inspections of mountainous roads.

## 6. Conclusions

In this study, we propose a novel UAV inspection system designed to examine pavement cracks on mountainous roads. Our system focuses on enhancing the autonomous capabilities in mountainous terrains by incorporating advanced embedded algorithms for route planning, autonomous navigation, and automatic crack detection. We have provided detailed information about the hardware design and introduced a real-time motion estimation algorithm that generates route planning and enables autonomous navigation in mountainous terrains. Simultaneously, the off-board computer conducts pavement crack detection in mountainous road environments. The UAV inspection system offers advantages such as mechanical simplicity, mobility, low weight, low cost, and real-time estimation. Therefore, our customized UAV inspection system is suitable for autonomous navigations in complex mountainous terrains, as well as pavement crack detection. To validate the system’s performance, we conducted several experiments to assess its accuracy, robustness, and efficiency. The experimental results demonstrate that our system accurately estimates the flight trajectory along the mountainous road for autonomous navigation, as well as conducts autonomous crack detection using our improved MRC-YOLOv8 for pavements, thus yielding more satisfactory results. In addition, a benchmark dataset for mountainous roads was also established and is open-source for the community.

In general, our UAV inspection system can provide significant assistance to traffic management authorities, rural planners, and infrastructure maintenance companies. It allows them to respond quickly to road issues and take timely measures, thereby improving road availability and safety. While UAV detection technology has advanced significantly, there is still a need for further research on mountainous road distress. In order to develop a comprehensive UAV road defect detection system, our future research will firstly concentrate on enhancing and modifying UAV hardware components to enhance flight stability and power durability, as well as look to enhance the capability to identify the cracks on mountain roads when within intricate road environments. We also plan to integrate more advanced deep learning architectures to enhance the performance of crack detection and segmentation. Furthermore, we acknowledge that real-world applications may be influenced by varying road conditions and environmental factors, which can affect detection performance. Therefore, we will explore the optimization and refinement of our model by applying it to diverse mountainous-terrain road scenarios.

## Figures and Tables

**Table 1:** Configuration of the main hardware components.

Hardware Device	Model	Specification
Visual Sensors (CMOS)	HIKVISION DS-E12 (Hikvision, Shenzhen, China) SPLIT-HDMI-CV2 (RunCam, Shenzhen, China)	RGB Resolution: 1920 × 1080; Pixels@30 FPS; Total Weight: 120 g; RGB Resolution: 1920 × 1080; Pixels@60 FPS; Total Weight: 22 g;
Mini-computer (Onboard)	NUC11 TNKi5 (Intel, Chandler, AZ, USA)	Operating System: Ubuntu18.04; Processor: Intel I5-1145G7 Memory: 8 GB, DDR4, 3200 MHz; Total Weight: 596 g;
Flight Controller	Pixhawk 6C, V2.4.8 (Chibei, Xuzhou, China)	Max input Voltage: 6 V; Power: 4.8 W; Operating temperature: −10°~+55°; Total Weight: 59.3 g;
Electronic Speed Control	Formula 32 bit 45 A ESC (Uangel, Shenzhen, China)	Persistent Current: 45 A; BEC: 10 V/2 A; Total Weight: 28.8 g;
GPS/IMU	WTG AHRS2 (WitMotion, Shenzhen, China)	Voltage: 3.3~5 V; Current: <40 mA; Angle Accuracy: 0.05;
Motors—4	Motor VELOX V2306 (Axisflying, Nanchang, China)	Power: 850 W; KV: 1950; Power ratio: 1.88; Total Weight: 128 g;
Battery	Gens ace XT60 4S (Grepow, Shenzhen, China)	Capacity: 8500 mAh; Voltage: 14.8 V; Total Weight: 680 g;

**Table 2:** Classification and description of road cracks in concrete material.

Having Damage with Cracks (Cracks)					No Damage	
Horizontal Cracks (HCs)	Vertical Cracks (VCs)	Oblique Cracks (OCs)	Block Cracks (BCs)	Pothole Cracks (PCs)	Expansion Joints (EJs)	No-Crack (NC) Background
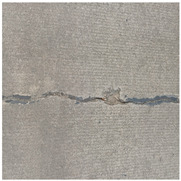	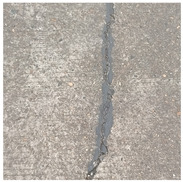	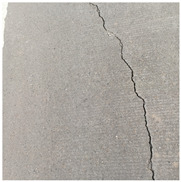	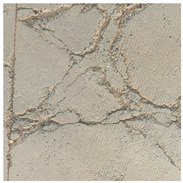	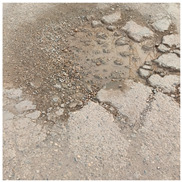	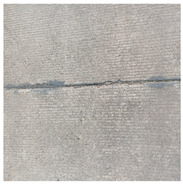	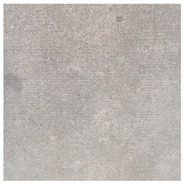

**Table 3:** Configuration of the experimental environment in a ground station.

Software	Configures	Matrix	Versions
Operating system	Ubuntu 20.4	Python	3.8
CPU	Intel(R) Xeon(R) Platinum 8352 V	PyTorch	1.11.0
GPU	RTX 4090 (24 GB)	CUDA	11.3
Memory	Samsung 64 GB		

**Table 4:** A comparison of the results following RMSE analysis (unit: m).

RMSE	X Axis RMSE	Y Axis RMSE	H Axis RMSE	Total Distance RMSE
The Sliding Window Method (SWM)	0.58	1.64	---	0.431
GPS only	0.51	1.95	0.571	0.544
The fusion of GPS/IMU	0.46	1.67	0.323	0.427
The final fusion results with SWM	0.46	1.52	0.219	0.339

**Table 5:** The results of the running performance with various models.

Models	Number of Parameters (×10^6^)	Training Duration (h)	Memory Consumption (MB)	*FPS* (f·s^−1^)
YOLO v5s	7.02	1.44	14.5	146.14
YOLO v8n	3.00	1.36	6.3	140.68
YOLO v9n	50.97	3.05	102.9	42.786
MRC-YOLOv8 (ours)	2.81	1.12	11.4	152.73

**Table 6:** Results of the overall accuracy in terms of the four evaluation metrics for four models (%).

Models	Precision (*P*)	Recall (*R*)	F1-Score	mAP
YOLO v5s	82.3	80.1	81.7	90.9
YOLO v8n	87.4	86.4	86.9	91.0
YOLO v9n	82.5	76.7	79.5	85.3
MRC-YOLOv8 (our)	90.7	89.8	87.4	92.3

**Table 7:** Detection accuracy results of the four models regarding the seven types of crack damage.

Models	mAP@0.5 (%)						F1-Score (%)					
	HCs	VCs	OCs	BCs	PCs	EJs	HCs	VCs	OCs	BCs	PCs	EJs
YOLO v5s	91.4	81.5	89.2	85.6	89.1	78.6	93.9	85.4	87.3	80.7	87.9	71.2
YOLO v8n	97.2	83.7	97.7	89.8	86.9	72.4	92.2	83.4	91.7	84.2	82.1	73.9
YOLO v9n	93.9	94.8	83.8	79.1	74.7	79.4	87.9	78.3	90.8	79.3	68.7	77.5
MRC-YOLOv8 (ours)	95.9	95.2	93.7	93.1	85.8	83.7	94.3	86.5	92.6	85.2	66.2	78.1

**Table 8:** Comparison of the pavement crack detection methods.

Systems	Main Sensors	Detection Methods	Advantages and Disadvantages
Sealing Prototype System [36]	Stereo Camera	Image Processing and Deep Learning	(1) Automated pavement crack detection; (2) Handheld work, labor-intensive, and inefficient;
Multi-function detection vehicles [37,38]	3D GPR/3D Laser Scanning	Transmitted Wave Method	(1) Highest accuracy detection; (2) High configuration and expensive;
UAV System [3,5,8,21,23,25]	Cameras	Image processing and Deep Learning	(1) Higher accuracy detection for cracks, with a mAP of >70%; (2) Commercial UAV—only for imagery data acquisition; (3) Offline pavement crack detection from imagery; (4) For urban roads and highways with flat and broader features;
UAV Autonomous Inspection System (Ours)	Cameras	Image processing and Deep Learning	(1) Higher accuracy detection for cracks, with a mAP of >80%; (2) A low-cost, customized UAV inspection system; (3) Automatic navigation and real-time crack detection; (4) For mountainous roads with curved and uneven terrain;

## Data Availability

The data that support the findings of this study are available from the corresponding authors upon reasonable request.
